# Complete genome sequence of a *Mycobacterium bovis* strain associated with a bovine tuberculosis outbreak on a cattle farm in Saskatchewan, Canada

**DOI:** 10.1128/mra.01090-24

**Published:** 2025-01-08

**Authors:** Olga Andrievskaia, Amalia Garceac, Dara Lloyd, Mirjana Savic, Marc-Olivier Duceppe

**Affiliations:** 1 Ottawa Laboratory Fallowfield, Canadian Food Inspection Agency, Ottawa, Ontario, Canada; University of Maryland School of Medicine, Baltimore, Maryland, USA

**Keywords:** bovine tuberculosis, Canada

## Abstract

Bovine tuberculosis is an important zoonotic infectious disease that presents a risk to human health, livestock, and wildlife. We report the complete genome sequence of a new *Mycobacterium bovis* strain that caused a bovine tuberculosis outbreak on a cattle farm in Saskatchewan, Canada, in 2023.

## ANNOUNCEMENT

*Mycobacterium bovis* is the most common causative agent of bovine tuberculosis (bTb), an important zoonotic disease (1). Currently, bTb has been mostly eradicated from Canadian livestock, except for the rare sporadic outbreaks (2). In 2023, one bTb outbreak was reported on a cattle farm in the province of Saskatchewan, Canada (3). Here, we present a complete genome sequence of the *M. bovis* strain affiliated with this outbreak to provide a reference for comparison with historical Canadian strains (4–6), and for future epidemiological investigations and disease surveillance. Lymph nodes with mycobacteriosis-compatible granulomatous lesions were collected for confirmatory diagnosis from cattle slaughtered as part of government-regulated bTb eradication activities, which were exempt from the animal experimentation ethics review process. Samples were processed for bacterial culture isolation and identification at the National Reference Laboratory for Bovine Tuberculosis, Canadian Food Inspection Agency. Tissue specimens were inoculated into several types of liquid and solid media (7) and incubated at 37°C. The *M. bovis* strain 2023/0113 was isolated from Modified Middlebrook with Malachite Green medium and taxonomically identified using biochemical and molecular tests (7) that included nitrate reduction and pyrazinamidase biochemical testing, species-specific PCRs, and partial 16S rDNA and *rpoB* gene sequencing. Genomic DNA was purified using MasterPure Gram Positive DNA Purification Kit (Lucigen, USA). The Illumina sequencing library was prepared with Nextera XT DNA Library Preparation Kit (Illumina Inc., USA), and 2,017,676 paired-end 250 bp reads were generated on Illumina MiSeq platform. The Nanopore sequencing library was prepared from the same DNA sample using Native Barcoding Kit SQK-NBD112.24 (Oxford Nanopore Technologies [ONT], UK) without shearing, and sequenced by a FLO-MIN112 flow cell on a MinION Mk1B device (ONT, UK) generating 139,898 reads with *N*_50_ of 4,637 bp. Nanopore reads were basecalled using Guppy v6.5.7 in super-accuracy mode (median *Q*-score of 17.4). Filtlong v0.2.1 (8) was used to filter out 5% bottom score reads. Nanopore reads were assembled with Flye v2.9.3 (9). The assembly was first polished with Nanopore reads using Medaka v1.11.3 (https://github.com/nanoporetech/medaka), and then with Illumina reads using a combination of Polypolish v0.6.0 (10) and pypolca v0.3.1 (11) with the “careful” flag after trimming/filtering with fastp v0.23.4 (12). The sequencing coverage depth was determined using Minimap2 v2.26 (13) and SAMtools v1.18 (14) for both Nanopore (120×) and Illumina (77×) reads. Default parameters of the bioinformatics software were used unless otherwise specified (https://github.com/duceppemo/BGA). Gene prediction and annotation were done using NCBI Prokaryotic Genome Annotation Pipeline v6.7 (15). The complete genome *M. bovis* 2023/0113 contains a single 4,350,333 bp chromosome with a BUSCO v5.7.1 (16) score 99.3%, a 65.64% G + C content, 4,082 predicted genes, 45 tRNAs, 2 clustered regularly interspaced short palindromic repeats. FastANI v1.33 (17) whole-genome comparison with a reference *M. bovis* strain AF2122/97 (18) generated an average nucleotide identity 99.98%.

According to the vSNP v3.20 (19) analysis, the *M. bovis* 2023/0113 differed by more than 50 single nucleotide polymorphisms from Canadian historical *M. bovis* strains (4–6), suggesting the introduction of a new strain into the country.

## Data Availability

Illumina and Nanopore sequencing reads are available in the NCBI Sequence Read Archive through accession numbers SRR28378595 and SRR29584401, respectively. The complete genome sequence of the *M. bovis* strain 2023/0113 is deposited in the NCBI GenBank under the accession number CP160832. The version described in this paper is the first version.
